# Establishment of a platform based on dual RPA combined with CRISPR/Cas12a for the detection of *Klebsiella pneumoniae* and its KPC resistance gene

**DOI:** 10.3389/fbioe.2024.1447963

**Published:** 2024-10-02

**Authors:** Meiying Tan, Xueli Yi, Chuan Liao, Zihan Zhou, Baoyan Ren, Lina Liang, Xuebin Li, Guijiang Wei

**Affiliations:** ^1^ Center for Medical Laboratory Science, Affiliated Hospital of Youjiang Medical University for Nationalities, Baise, China; ^2^ Baise Key Laboratory for Research and Development on Clinical Molecular Diagnosis for High-Incidence Diseases, Guangxi, China; ^3^ Key Laboratory of Research on Clinical Molecular Diagnosis for High Incidence Diseases in Western Guangxi, Guangxi, China; ^4^ Modern Industrial College of Biomedicine and Great Health, Youjiang Medical University for Nationalities, Baise, China; ^5^ Yaneng BlOscience (Shenzhen) Corporation, Guangxi, China

**Keywords:** carbapenem resistant *Klebsiella pneumoniae*, CRISPR/Cas12a, recombinase polymerase amplification, limit of detection, sensitivity

## Abstract

Carbapenem resistant *Klebsiella pneumoniae* (CRKP) can cause serious hospital- and community-acquired infections. Treatment for CRKP infection is limited, resulting in prolonged hospitalization and high consultation costs. The KPC genotype has the highest detection rate of CRKP, and its mortality rate is higher than the overall mortality rate of CRKP. However, traditional testing methods have disadvantages such as long time and reliance on complex and sophisticated instruments, which are not conducive to rapid screening for CRKP. Therefore, this study aimed to establish a detection platform for early screening of CRKP so that effective antimicrobial therapy could be administered promptly to prevent the widespread spread of CRKP. We integrated dual RPA with CRISPR/Cas12a to establish a dual platform for the detection of *K. pneumoniae* (*Kp*) rcsA-specific gene and KPC resistance gene. Four result reading methods were established, including fluorescence detection (FD), blue light irradiation detection (BLID), ultraviolet irradiation detection (UID), and lateral flow test strips (LFTS). For the rcsA gene, the LOD of FD was 1 × 10 pg/μL, and the other three methods could detect 1 × 10^1^ pg/μL of bacterial DNA. As for the KPC gene, four resultant readout methods were able to detect 1 × 10^2^ pg/μL of bacterial DNA. In 59 clinical strains tested, the dual RPA-CRISPR/Cas12a detection of the rcsA had 100% sensitivity, specificity, and accuracy compared to the culture method. Compared with the drug sensitivity test, the sensitivity of dual RPA-CRISPR/Cas12a detection for the KPC was 85.71%, the specificity was 100%, and the accuracy was 94.92%. In summary, our dual RPA-CRISPR/Cas12a platform proved to be rapid, precise, and convenient for the efficient detection of *Kp* with KPC in the laboratory or at the point of care.

## 1 Introduction

*Klebsiella pneumoniae* (*Kp*) is a Gram-negative conditionally pathogenic bacterium recognized as one of the primary pathogens responsible for hospital-acquired infections (Pitout et al., 2015; Tominaga, 2018). In recent times, the prevalent utilization of carbapenem antibiotics has led to an increase in carbapenem-resistant Enterobacteriaceae (CRE), particularly notable with carbapenem-resistant *Kp* (CRKP) (Aiesh et al., 2023; Hindiyeh et al., 2008; Xu et al., 2015). As drug resistance rates surge in CRE, infected individuals have high mortality rates and require longer treatment and higher consultation costs. Infection with CRE has now become a serious public health problem worldwide (Tompkins and van Duin, 2021; Zhang et al., 2018). Carbapenemases are usually categorized into class A, B, and D β-lactamases and *Kp* carbapenemase (KPC) belongs to class A carbapenemases, which effectively degrad carbapenem and β-lactam antibiotics (Cao et al., 2024). The main class A carbapenemases include KPC, SME, IMI, NMC, and GES, of which KPC was the most common and was mainly found on the plasmid of *Kp* (Queenan and Bush, 2007). The mortality rate of CRKP infection reached 27.5%–57% (Nakano et al., 2015), with pneumonia and bloodstream infections associated with a high risk of death (Zhang et al., 2018). Therefore, rapid identification of CRKP is crucial in clinical practice.

Currently, CRKP can be detected by phenotypic analysis and molecular diagnosis. Commonly used detection methods include the disk diffusion method, modified Hodge test, polymerase chain reaction (PCR), and sequencing analysis (Xu et al., 2022). However, these methods have disadvantages such as long testing times, expensive instruments, complicated operating procedures, and the need for specialized training, which are not conducive to their application in low-resource areas (Nakano et al., 2015). Numerous studies had devised isothermal amplification technology to enhance CRKP detection, such as recombinase aided amplification (Zhang et al., 2021), loop-mediated isothermal amplification (LAMP) (Nakano et al., 2015), LAMP-CRISPR/Cas12a (Xu et al., 2022), and LAMP-CRISPR/Cas13a (Liang et al., 2023). However, in these studies, only single resistance genes were detected and strain identification was also required. Furthermore, the LAMP was susceptible to generating dimers, which could result in false-positive outcomes (Dong et al., 2015). Hence, there is a need to devise a rapid and simple method for detecting both *Kp* and its KPC resistance gene.

Recombinase polymerase amplification (RPA) emerged as a novel isothermal amplification technology. It could be reacted at lower temperatures (37°C–42°C), had a short reaction time, did not require sophisticated and expensive instruments, was simple to operate, and had the potential to replace PCR (Liao et al., 2024). The clustered regularly interspaced short palindromic repeats (CRISPR) was first discovered in the immune system of bacteria and was a revolutionary gene editing technology (Jansen et al., 2002). With the discovery of related proteins of the CRISPR system, such as Cas9, Cas12a, Cas13a, and Cas14a, the CRISPR system was widely used in the field of nucleic acid detection. Cas12a was often used to detect DNA, which had both cis- and trans-cleavage activity (Li et al., 2018). Upon the formation of a ribonucleoprotein complex between Cas12a and CRISPR RNA (crRNA), it identified the protospacer adjacent motif site containing the “TTTN” motif and bound to double-stranded DNA (dsDNA), forming the Cas12a-crRNA-dsDNA complex. This complex initiated the targeted cleavage of dsDNA (Xiong et al., 2022). After targeted cleavage of dsDNA, the trans-cleavage activity of Cas12a was activated, allowing Cas12a to nonspecifically cleave arbitrary sequences of single-stranded DNA reporter (ssDNA) with fluorophore and quencher (Chen et al., 2018). RPA and Cas12a had been widely used to develop molecular detection platforms. For example, Chen et al. created the DETECTR platform based on isothermal amplification and Cas12a, which was successfully used for rapid detection of human papillomavirus (Chen et al., 2018). RPA combined with Cas12a was successfully applied to the detection of various pathogenic microorganisms, such as African swine fever virus and SARS-CoV-2 (Lin et al., 2022), *Vibrio parahaemolyticus* (Jiang et al., 2022), and *Pseudomonas aeruginosa* (Liu S. et al., 2023). For drug resistance detection, RPA-CRISPR/Cas12a was applied to detect Methicillin-resistant *Staphylococcus aureus* (Li et al., 2022), *Salmonella* virulence and drug resistance genes (Fu et al., 2022). However, there were relatively few studies applying RPA in combination with Cas12a to detect drug-resistant strains and especially fewer detection platforms that could detected bacterial identification genes and drug-resistance genes.

In this study, we established a dual detection platform utilizing both dual RPA and CRISPR/Cas12a and aimed at detecting *Kp* and its KPC resistance genes. This platform capitalized on the efficiency of RPA alongside the trans-cleavage activity of Cas12a. The platform contained four result readout methods: fluorescence detection (FD), blue light irradiation detection (BLID), ultraviolet irradiation detection (UID), and lateral flow test strips (LFTS) for the identification of *Kp* and the most common KPC resistance genes in different resource environments. Our platform provided a rapid clinical screening test for *Kp* with KPC, thereby enabling timely and effective treatment of patients and buying time for hospitals and communities to implement preventive and control measures to prevent the mass spread of *Kp* with KPC.

## 2 Materials and methods

### 2.1 Materials

Supplementary Table S1 presents the details of the standard strains utilized in this study. The Twist AmpTM Basic Kit was procured from Twist DX Co., Ltd. (Hertfordshire, U.K.), while Cas12a and NEBuffer r2.1 were sourced from New England Biolabs (Guangzhou, China). Additionally, the 2×Es Taq MasterMix for PCR was obtained from Cwbio Co., Ltd. (Guangxi, China). TIANamp Bacterial DNA kit was purchased from TIANGEN Biochemical Technology Co. Cas12/13 nucleic acid test strips were purchased from Beijing Baoying Tonghui Biotechnology Co. Gel imaging and UV irradiation were performed using an Amersham Imager 600 (GE Healthcare, USA). The oligonucleotide sequences used in this study were synthesized by Nanning GenSys Biotechnology Co., Ltd. (Nanning, China). All oligonucleotide sequences are shown in Supplementary Table S2.

### 2.2 DNA extraction

All standard strains were resuscitated and cultured on blood plates. Afterward, individual colonies were picked to extract the standard strain DNA according to the steps of the TIANamp Bacterial DNA kit. DNA concentration was measured using a spectrophotometer, and the OD260/280 value of the qualifying sample should be between 1.8 and 2.0. The collected clinical strains were stored at −80°C using skimmed milk medium. DNA from clinical strains was also extracted using the TIANamp Bacterial DNA kit. Bacterial DNA genomes that had not yet been used were stored at −80°C.

### 2.3 Primers and crRNA design

The sequences of rcsA and KPC genes were obtained from NCBI (https://www.ncbi.nlm.nih.gov/), and four pairs of RPA primers were designed using Primer Premier five targeting rcsA and KPC genes, respectively. The specificity of the RPA primers was initially evaluated by “Primer-BLAST” on NCBI. The optimal RPA primers were then further screened using 2% agarose gel electrophoresis. The crRNA for the rcsA and KPC genes were designed by the CRISPR online tool (http://www.rgenome.net/cas-designer) at the amplicon in the middle of the optimal primers, respectively, and two crRNAs were designed for each gene. The optimal crRNA was screened by comparing the fluorescence intensity generated by Cas12a cleavage.

### 2.4 Establishment and optimization of dual RPA amplification system

The volume of the double RPA was 25 μL, including 14.75 μL of buffer, 3 μL of ddH_2_O, 4 μL of primers (2 μL for each primer pair), 2 μL of template DNA, and 1.25 μL of magnesium acetate. The dual RPA amplification system was optimized to improve the amplification efficiency of both genes. Firstly, the two pairs of primer ratios were optimized, and the concentration ratios of rcsA and KPC primers were set to 2 μM:10 μM, 4 μM:10 μM, 6 μM:10 μM, 8 μM:10 μM, 10 μM:10 μM, 10 μM:8 μM, 10 μM:6 μM, 10 μM:4 μM, and 10 μM:2 μM, and the optimal ratio was then screened by comparing the results of agarose gel experiments. In addition, the amplification time of the dual RPA was optimized by setting the amplification time to 10 min, 15 min, 20 min, 25 min, and 30 min, and the amplification products were also analyzed by 2% agarose gel electrophoresis to determine the optimal RPA time.

### 2.5 Establishment of CRISPR/Cas12a cleavage system

The volume of the Cas12a system was 20 μL, including 2 μL NEBuffer r2.1 (10×), 10 μL ddH_2_O, 2 μL ssDNA (10 μM), 2 μL crRNA (1 μM), 2 μL Cas12a (1 μM), and 2 μL template DNA. In this study, CRISPR/Cas12a cleavage results could be analyzed in four ways. The FD was performed using a qPCR instrument to read fluorescence at 1-min intervals for 30 min at 37°C. In addition, following a 30-min incubation at 37°C, the Cas12a system could be exposed to UV or blue light, allowing for the visual observation of outcomes with the unaided eye. For the LFTS method, following completion of the Cas12a reaction, it was essential to supplement with ddH_2_O to reach a total volume of 50 μL. Subsequently, the test strip was inserted into the Cas12a system, and results were interpreted within 10 min.

### 2.6 Optimization of the CRISPR/Cas12a system

Using the *Kp* with KPC standard strain (ATCC1705) as the target and ddH_2_O as the negative control, the concentration of ssDNA and the ratio of crRNA and Cas12a were optimized to fully utilize the performance of the detection platform. The ssDNA concentrations were set to 500 nM, 750 nM, 1,000 nM, and 1250 nM. The volume ratio of crRNA and Cas12a (original concentrations of both crRNA and Cas12a were 1 μM) were set to 1 μL:1 μL, 1 μL:2 μL, 1 μL:3 μL, 2 μL:1 μL, 2 μL:2 μL, 2 μL:3 μL, 3 μL:1 μL, 3 μL:2 μL, and 3 μL:3 μL. The optimal ssDNA concentration and crRNA/Cas12a were screened by comparing the fluorescence values, signal-to-noise ratios F_K_/F_0_ (F_K_, *Kp*-excited fluorescence, F_0_, ddH_2_O-excited fluorescence), and the results of BLID and UID.

### 2.7 Specificity and limit of detection (LOD) of dual detection platform

We designed 15 reactions and one negative control (ddH_2_O) to validate the specificity of the dual RPA-CRISPR/Cas12a platform using either a single standard strain or a mixture of standard strains as targets. Moreover, the *Kp* with KPC standard strain (ATCC1705) was used as a target to determine the LOD of this platform. After extracting the DNA, the DNA concentration of 1 × 10^3^ pg/μL was measured with a spectrophotometer. Bacterial DNA concentrations ranging from 1 × 10^3^ pg/μL to 1 × 10^−3^ pg/μL were obtained by several 10-fold dilutions using ddH_2_O as the diluent. Finally, different concentrations of DNA were used as targets to evaluate the LOD of dual RPA-CRISPR/Cas12a.

### 2.8 Verification of clinical strain detection capability

Tovalidate the clinical application of the platform, 59 clinical strains were collected from the clinical laboratory of The Affiliated Hospital of Youjiang Medical University for Nationalities. PCR and dual RPA-CRISPR/Cas12a were used to detect all strains separately, with ddH_2_O as a negative control. The detection performance of the dual RPA-CRISPR/Cas12a platform was assessed by comparing the results of all methods. The PCR reaction comprised a volume of 25 μL, consisting of 0.4 μL primers (forward or reverse primers), 12.5 μL of 2×Es Taq MasterMix, 2 μL of template DNA, with the remaining volume adjusted to 25 μL using ddH_2_O. The primers used for PCR were shown in Supplementary Table S2.

### 2.9 Statistical analysis

The data was statistically analyzed using IBM SPSS Statistics 24. Fluorescence detection results were compared by Student’s T-test, with significance determined at *p* < 0.05. Kappa was employed to evaluate methodological consistency.

## 3 Result

### 3.1 The workflow of the dual RPA-CRISPR/Cas12a platform

**FIGURE 1:**
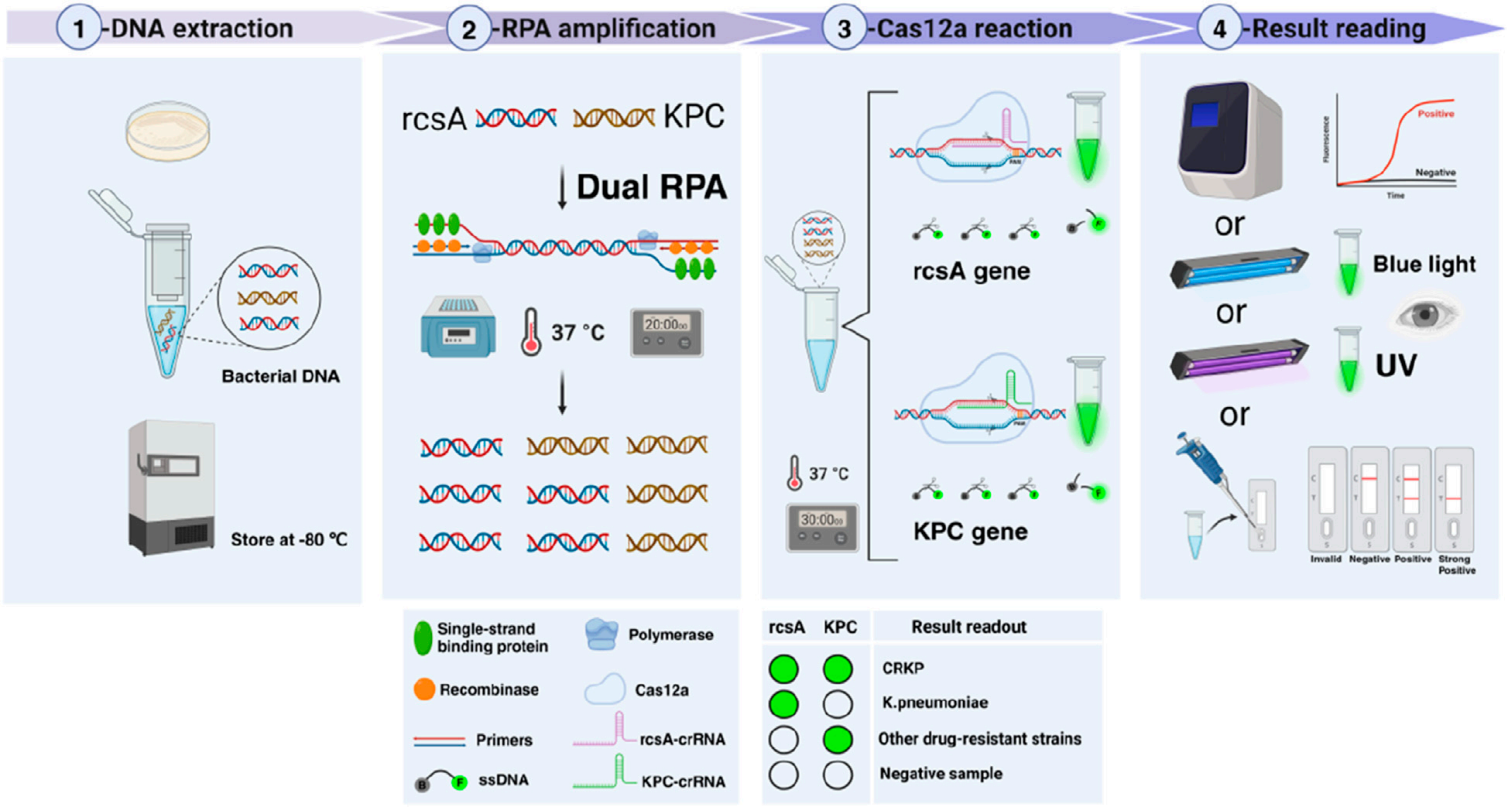
Workflow of the dual RPA-CRISPR/Cas12a detection platform. Firstly, the DNA of the bacteria was extracted and the unused DNA was stored at −80°C. Secondly, dual RPA amplification of the extracted DNA was performed at 37°C for 20 min to obtain a large number of amplification products. Thirdly, the dual RPA amplification products were added to two tubes of CRISPR/Cas12a system containing different crRNAs and reacted at 37°C for 30 min. Finally, the detection results were read by four methods including FD, BLID, UID, and LFTS.

The detailed workflow of the dual RPA-CRISPR/Cas12a detection platform is shown in Figure 1. Firstly, the DNA of the bacteria was extracted and the unused DNA was stored at −80°C. Secondly, the dual RPA amplification reaction could be completed by incubation at 37°C for 20 min. This reaction was driven by components including primers, recombinase, single-stranded binding protein, and polymerase. Thirdly, the dual RPA amplification products were added to two EP tubes containing the Cas12a reaction system, and the two systems were identical in composition except that the crRNAs were different (rcsA-crRNA and KPC-crRNA, respectively). After 30 min of reaction at 37°C, the results could be read by different methods. This study included an FD method for detecting fluorescence by qPCR instrument, BLID, and UID methods for visualizing the results, and an LFTS method that could be applied to POCT.

### 3.2 Screening of primers and crRNA

**FIGURE 2:**
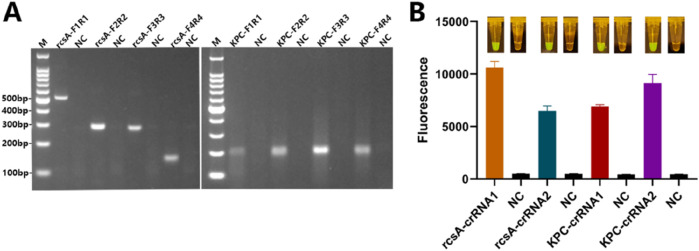
RPA primers and crRNA screening. **(A)** Agarose gels after RPA of different primer pairs for rscA and KPC genes. NC - negative control; M - Marker. **(B)** Mean +standard deviation of fluorescence intensities of different crRNAs for rscA and KPC genes after Cas12a cleavage and spectroscopic readout. The inserted images show the macroscopically visible flouresence after blue light irradiation.

Four pairs of RPA primers were designed using *Kp* rcsA-specific genes and the most common KPC resistance genes as targets, respectively. As shown in Figure 2A, rcsA-F2R2, and KPC-F3R3 primers had the best amplification efficiency and did not show non-specific amplification bands, so rcsA-F2R2 and KPC-F3R3 were used for dual RPA amplification in the subsequent experiments. Subsequently, two crRNAs were designed at the amplicons in the middle of each of the rcsA-F2R2 and KPC-F3R3. As shown in Figure 2B, the reaction using the rcsA-crRNA1 and KPC-crRNA2 produced stronger fluorescence values and good visualization, so rcsA-crRNA1 and KPC-crRNA2 were selected as the best crRNA.

### 3.3 Optimization of the dual detection platform

To maximize the detection efficiency of the dual RPA-CRISPR/Cas12a platform, the dual RPA and CRISPR/Cas12a systems were separately optimized. The dual RPA was mainly optimized for the ratio of the two primer pairs and the amplification time. As shown in Supplementary Figure S1A, the amplification efficiency of both genes was better when the concentration ratio of rcsA-F2R2 and KPC-F3R3 was 4 μM:10 μM. The result was shown in Supplementary Figure S1B, when the dual RPA amplification was performed for 20 min, a sufficient amount of amplified product was obtained. To achieve a rapid and efficient detection balance, we selected 20 min as the subsequent RPA amplification time. Therefore, the primer concentration of dual RPA was optimized as 4 μM:10 μM (rcsA-F2R2:KPC-F3R3), and the amplification time was 20 min.

**FIGURE 3:**
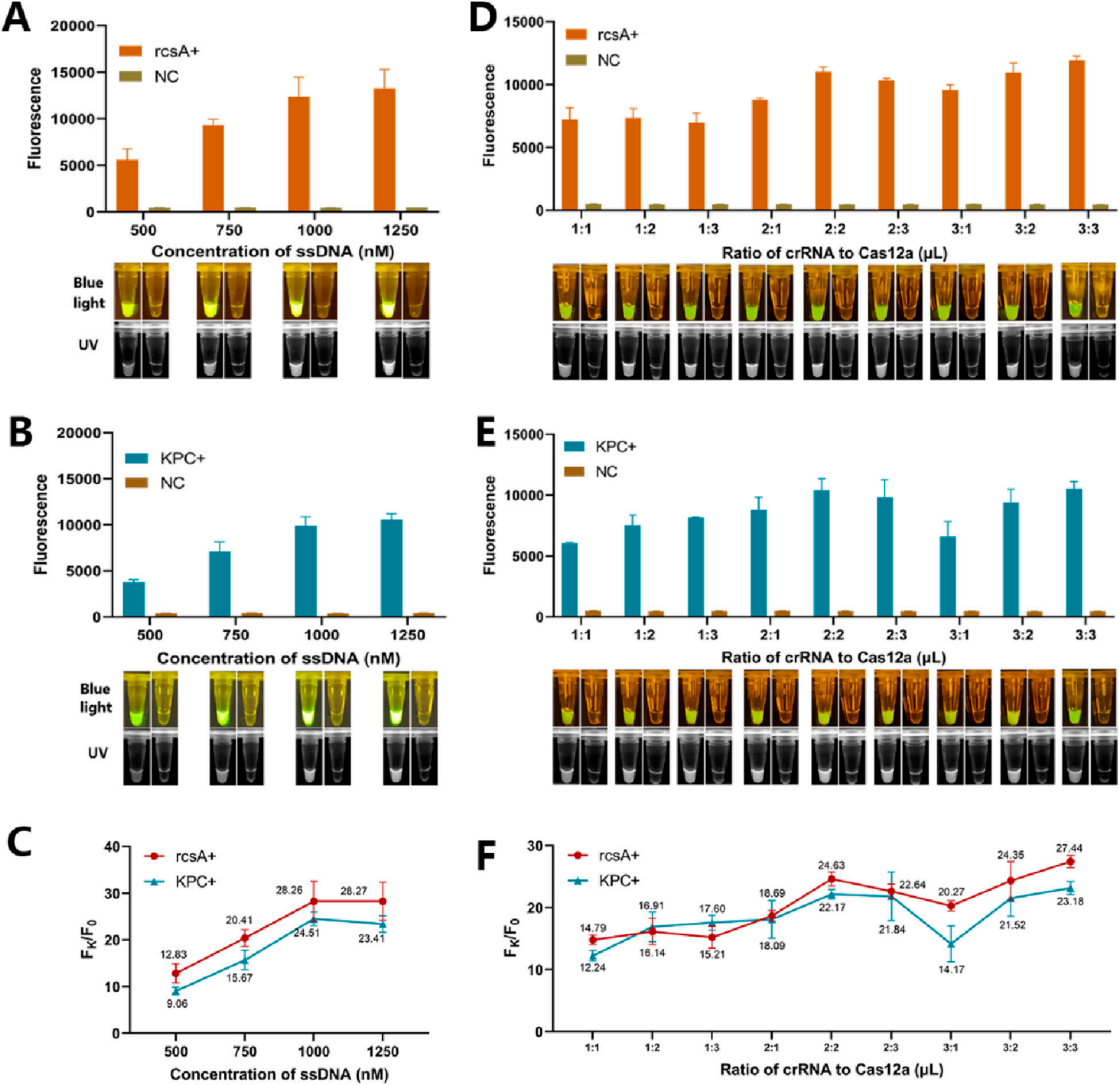
Optimization of the CRISPR/Cas12a cleavage system. **(A)** Mean +standard deviation of fluorescence intensities of different ssDNA concentration for rcsA genes after Cas12a cleavage and spectroscopic readout. **(B)** Mean +standard deviation of fluorescence intensities of different ssDNA concentration for KPC genes after Cas12a cleavage and spectroscopic readout. **(C)** Ratio of the fluorescence value F_k_ produced by the rcsA/KPC gene to the background fluorescence value F_0_ produced by ddH_2_O at different ssDNA concentrations. **(D)** Mean +standard deviation of fluorescence intensities of different crRNA: Cas12a for rcsA genes after Cas12a cleavage and spectroscopic readout. **(E)** Mean +standard deviation of fluorescence intensities of different crRNA: Cas12a for KPC genes after Cas12a cleavage and spectroscopic readout. **(F)** Ratio of the fluorescence value F_k_ produced by the rcsA/KPC gene to the background fluorescence value F_0_ produced by ddH_2_O at different crRNA: Cas12a. In all images, the inserted color image show the macroscopically visible flouresence after blue light irradiation and the black and white image show the macroscopically visible flouresence after UV irradiation.

To screen the optimal ssDNA concentration for dual RPA-CRISPR/Cas12a and to make the signals generated by cleavage more easily identified, the ssDNA concentration was set from 500 nM to 1,250 nM in this study, and four sets of Cas12a reactions were carried out using the dual RPA amplification products, respectively. As shown in Figures 3A, B, for the rcsA gene, when the ssDNA concentration was 1,000 nM, it could produce sufficiently strong fluorescence intensity and the F_K_/F_0_ was not much different from 1,250 nM (Figure 3C), considering the cost of reagents, 1,000 nM of ssDNA was chosen for the subsequent experiments. For the KPC gene, sufficiently strong fluorescence values could be produced when the ssDNA concentration was 1,000 nM, and F_K_/F_0_ was maximum (Figure 3C). Therefore, 1,000 nM of ssDNA was selected for subsequent experiments for both rcsA and KPC genes. Under the above optimal conditions, nine different sets of crRNA and Cas12a ratios were designed to optimize crRNA/Cas12a. As shown in Figures 3D, E, the Cas12a system for detecting both genes produced sufficiently strong fluorescence signals when the crRNA/Cas12a was 2 μL:2 μL, and the visual results of both BLID and UID were good. As shown in Figure 3F, when crRNA/Cas12a was 2 μL:2 μL, F_K_/F_0_ was second only to 3 μL:3 μL, and 2 μL:2 μL was chosen for subsequent experiments to save reagent costs.

### 3.4 The specificity and the LOD of the dual detection platform

**FIGURE 4:**
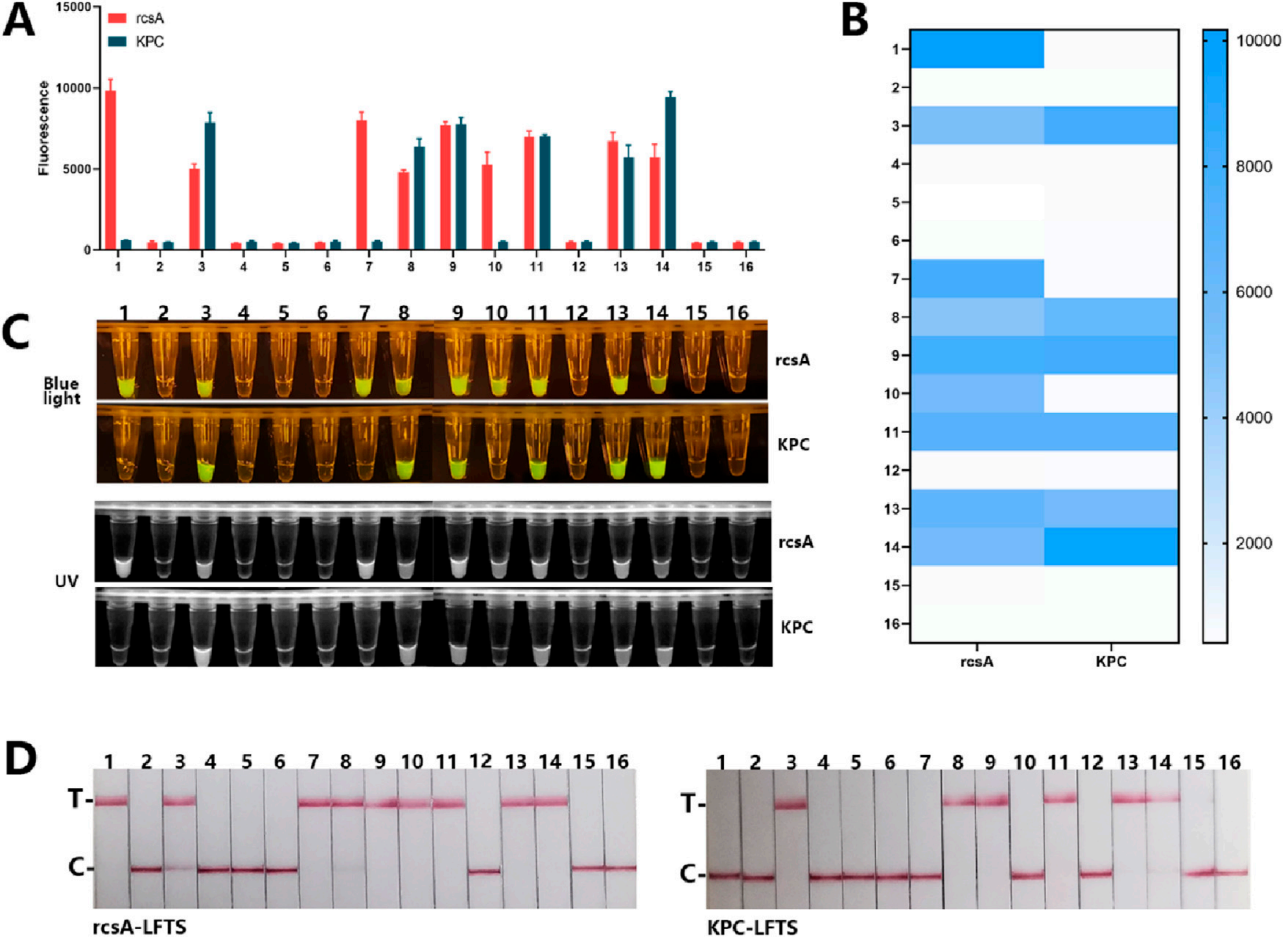
The specificity of dual RPA-CRISPR/Cas12a detection platform. **(A)** Mean +standard deviation of fluorescence intensity after Cas12a cleavage of 16 different targets. **(B)** Heatmap of the specificity results of the FD method for rcsA and KPC genes. **(C)** The macroscopically visible flouresence images of blue light and UV irradiation after Cas12a cleavage of 16 different targets. **(D)** Results of LFTS method after Cas12a cleavage of 16 different target. (1. *Kp*, 2. *E.coli*, 3. *Kp* with KPC, 4. *S.aureus*, 5. *S.typhimurium*, 6. *A.baumannii*, 7. *E.coli* + *Kp*, 8. *S.typhimurium* + *Kp* with KPC, 9. *S.aureus* + *Kp* with KPC, 10. *A.baumannii* + *Kp*, 11. *S.typhimurium* + *Kp* with KPC, 12. *E.coli* + *S.pneumoniae*, 13. *E.coli* + *S.pneumoniae* + *Kp* with KPC, 14. *S.aureus* + *A.baumannii* + *Kp* with KPC, 15. *E.coli* + *A.baumannii* + *S.pneumoniae*, 16. negative control).

**FIGURE 5:**
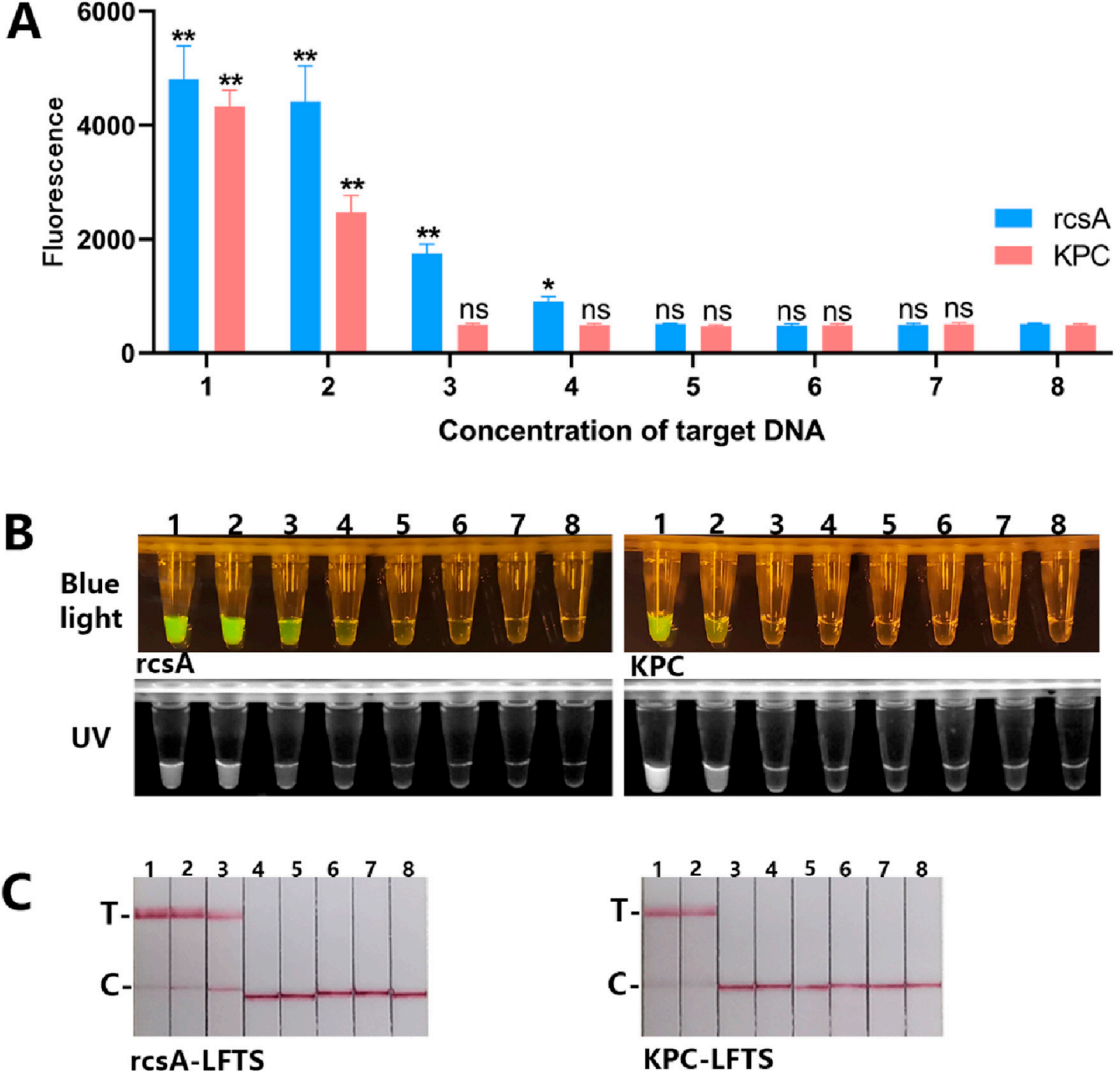
The LOD of the dual RPA-CRISPR/Cas12a platform. **(A)** Mean +standard deviation of fluorescence intensities of different rcsA and KPC genes concentration after Cas12a cleavage and spectroscopic readout. **(B)** The macroscopically visible flouresence images of blue light and UV irradiation after Cas12a cleavage of different targets concentration. **(C)** Results of LFTS method after Cas12a cleavage of different target concentration. (1. 1 × 10^3^ pg/μL; 2. 1 × 10^2^ pg/μL; 3. 1 × 10^1^ pg/μL; 4. 1 × 10° pg/μL; 5. 1 × 10^−1^ pg/μL; 6. 1 × 10^−2^ pg/μL; 7. 1 × 10^−3^ pg/μL; 8. negative control).

To validate the specificity of the detection platform, we used the established dual RPA-CRISPR/Cas12a platform to detect 15 targets containing DNA from one or more standard strains. As shown in Figure 4, the four methods showed positive results only when testing targets containing *Kp* or KPC resistance genes. When the target contained non-resistant *Kp*, only the rcsA detection system showed positive results. When the target was one or more other standard strains that did not produce KPC, no positive results were produced. *Kp* with KPC genomic DNA underwent a series of tenfold dilutions ranging from 1 × 10^3^ pg/μL to 1 × 10^−3^ pg/μL. These dilutions were utilized to validate the LOD of the dual RPA-CRISPR/Cas12a platform. As shown in Figure 5, for the rcsA gene, the LOD of FD was 1 × 10° pg/μL. The LOD of BLID, UID, and LFTS was 1 × 10^1^ pg/μL. As for the KPC gene, the LOD of the FD, BLID, UID, and LFTS methods was 1 × 10^2^ pg/μL.

### 3.5 Verification of clinical strain detection capability

**FIGURE 6:**
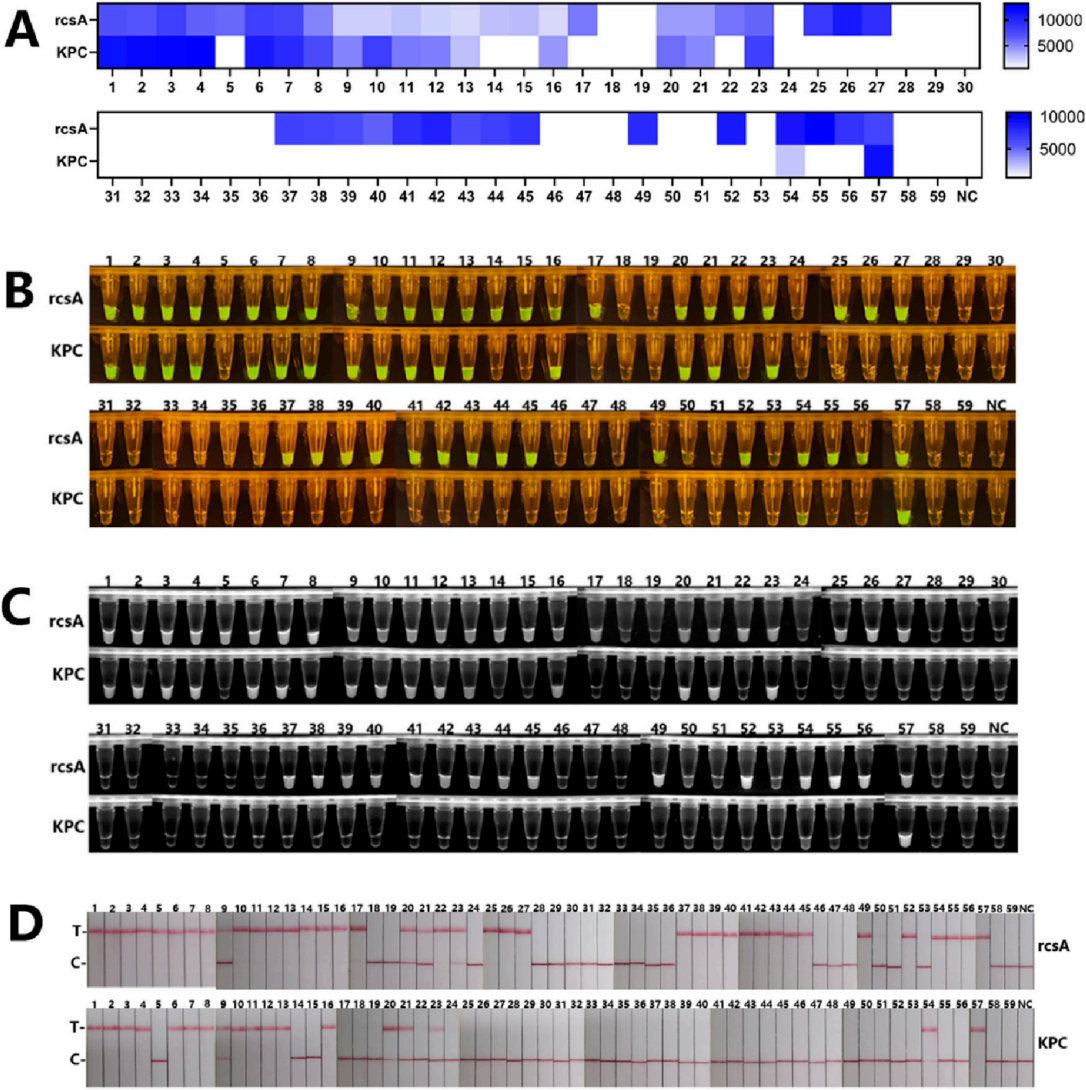
The results of clinical strains detection by the dual RPA-CRISPR/Cas12a platform. **(A)** Fluorescence values of 59 clinical samples with readout after Cas12a cleavage. **(B)** The macroscopically visible flouresence images of blue light irradiation after Cas12a cleavage of 59 clinical samples. **(C)**The macroscopically visible flouresence images of UV irradiation after Cas12a cleavage of 59 clinical samples. **(D)** Results of LFTS method after Cas12a cleavage of 59 clinical samples.

We conducted dual RPA-CRISPR/Cas12a and PCR detection on 59 clinical strains identified by the culture method to validate the clinical applicability of the platform. The results of the PCR assay were shown in Supplementary Figure S2, 39 strains were detectable for the rcsA gene and confirmed to be *Kp*, of which 20 strains were detectable for the KPC gene. The other 20 clinical strains were negative for both genes. As shown in Figure 6, 39 strains were detected to contain the rcsA gene by four result readout methods, of which 18 strains produced KPC, and the other 20 strains were free of both rcsA and KPC genes.

## 4 Discussion

CRKP is a highly drug-resistant bacterium that belongs to a variant of *Kp*. The World Health Organization identified CRKP as one of the key priority pathogens for which research and development of novel and effective antibiotic therapies were urgently needed (Ortiz-Cartagena et al., 2023). If bacteria become resistant to carbapenem antibiotics, there are very few drug options for CRKP infections. A study showed that the combined mortality rate of CRKP patients was 42.14%. Whereas, the mortality rate of KPC-resistant *Kp* was higher than the combined total mortality rate (47.66% vs 42.14%), which might be attributed to its more aggressive nature (Xu et al., 2017). *Kp* with KPC is highly transmissible and can lead to multiple infections and outbreaks in hospitals. Early detection of *Kp* with KPC is important for guiding clinical treatment and controlling infection and transmission of drug-resistant strains.

Currently, the main detection methods for CRKP are the disk diffusion method, the modified Hodge test, and the PCR method (Xu et al., 2024). However, these conventional methods often rely on sophisticated instrumentation and required specialized training of operators. These limitations restrict the use of many traditional methods in primary health units and low-resource areas. RPA was an isothermal amplification technology that could be reached at 37°C–42°C and did not require expensive instrumentation, but there was a certain amount of nonspecific amplification with RPA (Gong et al., 2022), so further signal amplification is necessary. In the Cas12a system, secondary recognition of the target by crRNA can further enhance the specificity of the assay, but the Cas12a system alone for nucleic acid detection has a low detection efficacy (Chen et al., 2022). Therefore, combining RPA with CRISPR/Cas12a to compensate for their respective shortcomings will greatly improve the performance of the assay.

In this study, we integrated dual RPA and Cas12a to establish a detection platform for *Kp* with KPC. The platform simultaneously amplified the *Kp* rcsA-specific and KPC resistance genes by dual RPA, and then divided into two tubes for CRISPR/Cas12a reaction. If two tubes were used for simultaneous CRISPR/Cas12a cutting, bacterial identification, and KPC resistance gene detection could be performed at the same time. Our detection platform could detect the *Kp* identification gene rcsA and the drug resistance gene KPC, with no cross-reactivity to other clinical strains and high specificity. We established four methods for reading results, including FD, BLID, UID, and LFTS. Among them, the BLID, UID, and LFTS methods could observe the results with the naked eye, simplifying the instrument equipment. The FD could detect specific values and was more advantageous in terms of sensitivity. For the rcsA, the FD could detect as low as 1 × 10°pg/μL, which was ten times higher than the BLID, UID, and LFTS methods (1 × 10^1^ pg/μL). The sensitivity of this platform for detecting the KPC gene was poorer than the rcsA gene, probably because the primers for the rcsA gene were amplified more efficiently than those for the KPC gene. For the KPC assay, the LOD of all four result reading methods was 1 × 10^2^ pg/μL, which could satisfy the detection of routinely extracted bacterial DNA concentration. As shown in Table 1, compared with the culture method, the dual platform had 100% sensitivity specificity, and accuracy. As shown in Table 2, compared with the drug sensitivity test, the dual platform had a sensitivity of 85.71%, 100% specificity, and 94.92% accuracy. The Kappa value was 0.885 and the two methods were highly consistent (*p > 0.05*). The dual detection platform established in this study was expected to be a screening platform for CRKP, especially the three visualization methods were very beneficial for screening CRKP in low-resource areas.

**TABLE 1:** The results of rcsA gene detection using dual RPA-CRISPR/Cas12a platform and culture method.

	Culture method			Sensitivity (%)	Specificity (%)	Accuracy (%)	Kappa	*P*
		+	—					
FD	+	39	0	100	100	100	1.000	1.000
	—	0	20					
BLID	+	39	0	100	100	100	1.000	1.000
	—	0	20					
UID	+	39	0	100	100	100	1.000	1.000
	—	0	20					
LFTS	+	39	0	100	100	100	1.000	1.000
	—	0	20					

**TABLE 2:** The results of KPC gene detection using dual RPA-CRISPR/Cas12a platform and drug sensitivity test.

	Culture method			Sensitivity (%)	Specificity (%)	Accuracy (%)	Kappa	*P*
		+	—					
FD	+	18	0	85.71	100	94.92	0.885	0.250
	—	3	38					
BLID	+	18	0	85.71	100	94.92	0.885	0.250
	—	3	38					
UID	+	18	0	85.71	100	94.92	0.885	0.250
	—	3	38					
LFTS	+	18	0	85.71	100	94.92	0.885	0.250
	—	3	38					

However, our detection platform currently still had some limitations. First, the platform mainly detected the most common KPC genotypes in CRKP and did not include other types of carbapenem resistance genes. In addition, the use of dual tubes to detect rcsA and KPC genes separately during the Cas12a reaction phase increases costs, and repeated uncapping and pipetting may generate aerosol contamination, leading to false-positive results. In the future, we will further optimize the current strategy by modifying the oligonucleotides of the Cas12a cleavage system (Han et al., 2023) or employing a combination of Cas12a and Cas13a methods (Liu Y. et al., 2023; Tian et al., 2022) to improve towards a one-pot method for multiple detection, thereby reducing experimental operations and aerosol contamination. Meanwhile, more rapid and simple bacterial DNA extraction methods, such as boiling method, were tried to be used for bacterial DNA extraction, thus simplifying the whole detection process.

## 5 Conclusion

In summary, this study firstly detected *Kp* and its KPC resistance gene by the detection platform based on dual RPA-CRISPR/Cas12a. Compared to traditional detection methods including the disk diffusion method, modified Hodge test, PCR, and so on, the dual RPA-CRISPR/Cas12a detection platform has the advantages of fast, low-cost, easy operation, and high sensitivity and specificity.

In addition, this study boldly attempted to detect RPA-CRISPR/Cas12a signals using four methods including FD, BLID, UID, and LFTS simultaneously. The outcome of our study provided important evidence in this research domain and allowed clinical technicians to choose the most suitable detection method under diffrent laboratory environments. In the future, the dual RPA-CRISPR/Cas12a platform could be widely used for rapid screening of *Kp* with KPC, enabling patients to receive timely treatment and thus preventing the widespread spread of *Kp* with KPC.

## Data Availability

The original contributions presented in the study are included in the article/Supplementary Material, further inquiries can be directed to the corresponding authors.
