# Supplementary material for: Establishment of a platform based on dual RPA combined with CRISPR/Cas12a for the detection of Klebsiella pneumoniae and its KPC resistance gene
Source: Front Bioeng Biotechnol. 2024 Oct 2;12:1447963. doi: 10.3389/fbioe.2024.1447963 (PMC11480703; doi:10.3389/fbioe.2024.1447963)
Supplement: Supplementary file 1 [file DataSheet1.docx]

**Supplementary Material**

**A novel detection platform based on dual RPA combined with CRISPR/Cas12a for the detection of *Klebsiella pneumoniae* and its KPC resistance gene**

Meiying Tan^a,b,c^, , Xueli Yi^a,b,c^, Chuan Liao^a,b,c^, Zihan Zhou^a,b,c^, Baoyan Ren^d,e^, Lina Liang^a,b,c^*, Xuebin Li^d^*, Guijiang Wei^a,b,c,d^*

^a^Center for Medical Laboratory Science, Affiliated Hospital of Youjiang Medical University for Nationalities, Guangxi, 533000, China

^b^Baise Key Laboratory for Research and Development on Clinical Molecular Diagnosis for High-Incidence Diseases, Guangxi, 533000, China

^c^Key Laboratory of Research on Clinical Molecular Diagnosis for High Incidence Diseases in Western Guangxi, Guangxi, 533000, China

^d^Modern Industrial College of Biomedicine and Great Health, Youjiang Medical University for Nationalities, Guangxi, 533000, China.

^e^Yaneng BlOscience (Shenzhen) Corporation, Guangdong, 518118, China.

*Corresponding author: Guijiang Wei (email: weiguijiang2021@163.com); Xuebin Li (email: 00025@ymun.edu.cn);Lina Liang (email: 247949108@qq.com);

**TABLE S1** Information on all standard strains in this study

| Name | Number | Source |
| --- | --- | --- |
| *Kp* | ATCC13883 | American Type Culture Collection |
| *Kp* with KPC | ATCC1705 | American Type Culture Collection |
| *E.coli* | ATCC25922 | American Type Culture Collection |
| *S.typhimurium* | ATCC14028 | American Type Culture Collection |
| *S.aureus* | ATCC25923 | American Type Culture Collection |
| *E.faecalis* | ATCC35667 | American Type Culture Collection |
| *S.pneumoniae* | ATCC49619 | American Type Culture Collection |
| *A.baumannii* | ATCC19609 | American Type Culture Collection |

| **Name** | **Sequence(5’- 3’)** | **Length (bp)** | **Amplicon size (bp)** |
| --- | --- | --- | --- |
| rcsA-F1 | TTGACGGGATATCTGACCAGTCGGGGAATT |  | 514 |
| rcsA-R1 | ACCCGGCGACGCTGTTTGTTATCTTTATGT |  |  |
| rcsA-F2 | ACCCGGCGACGCTGTTTGTTATCTTTATGT | 30 | 286 |
| rcsA-R2 | GATACCGTCTTCGCTTTGATGTTCATTTGC | 30 |  |
| rcsA-F3 | CGGAAGAATCTGCTAATCAGTTCAAAATCG | 30 | 279 |
| rcsA-R3 | ATGATAAATCACCTGCTTATTATGCGTTTG | 30 |  |
| rcsA-F4 | GTACGGAAGAATCTGCTAATCAGTTCAAAA | 30 | 141 |
| rcsA-R4 | TGATTCTGTTTTACTCAGTGACAATGTCGG | 30 |  |
| KPC-F1 | GTTCCGTCTGGACCGCTGGGAGCTGGAG | 28 | 187 |
| KPC-R1 | GATGCGGTGGTTGCCGGTCGTGTTTCCC | 28 |  |
| KPC-F2 | GTCTGGACCGCTGGGAGCTGGAGCTGAAC | 29 | 184 |
| KPC-R2 | CGGATGCGGTGGTTGCCGGTCGTGTTTC | 28 |  |
| KPC-F3 | CGTTCCGTCTGGACCGCTGGGAGCTGGA | 28 | 186 |
| KPC-R3 | TGCGGTGGTTGCCGGTCGTGTTTCCCTT | 28 |  |
| KPC-F4 | CGTCTGGACCGCTGGGAGCTGGAGCTGAA | 29 | 181 |
| KPC-R4 | TGCGGTGGTTGCCGGTCGTGTTTCCCTTT | 29 |  |
| rcsA-crRNA1 | UAAUUUCUACUAAGUGUAGAUCCGACAUUGUCACUGAGUAA | 41 |  |
| rcsA-crRNA2 | UAAUUUCUACUAAGUGUAGAUCUCAGUGACAAUGUCGGUAA | 41 |  |
| KPC-crRNA1 | UAAUUUCUACUAAGUGUAGAUCGUCACGGCGCGCGGCGAUG | 41 |  |
| KPC-crRNA2 | UAAUUUCUACUAAGUGUAGAUUAAGCUUUCCGUCACGGCGC | 41 |  |
| rcsA-PCR-F | TAAACCTACTATTATTATCGCCCG | 24 | 147 |
| rcsA-PCR-R | ATATCCCGTCAATCCCAACC | 20 |  |
| KPC-PCR-F | GCCGTCTAGTTCTGCTGTCT | 20 | 141 |
| KPC-PCR-R | CGGTATCCATCGCGTACACA | 20 |  |

**TABLE S2** Nucleic acid sequences used in this study


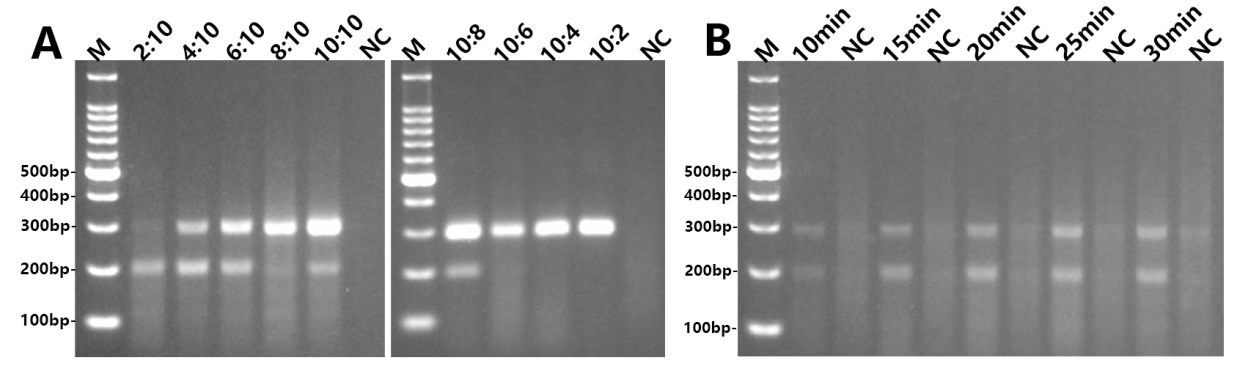


**FIGURE S1** Optimisation of RPA amplification system. (A) Optimisation of rcsA-F2R2 and KPC-F3R3 primer concentration ratios. (B) Optimisation of dual RPA amplification time.

**FIGURE
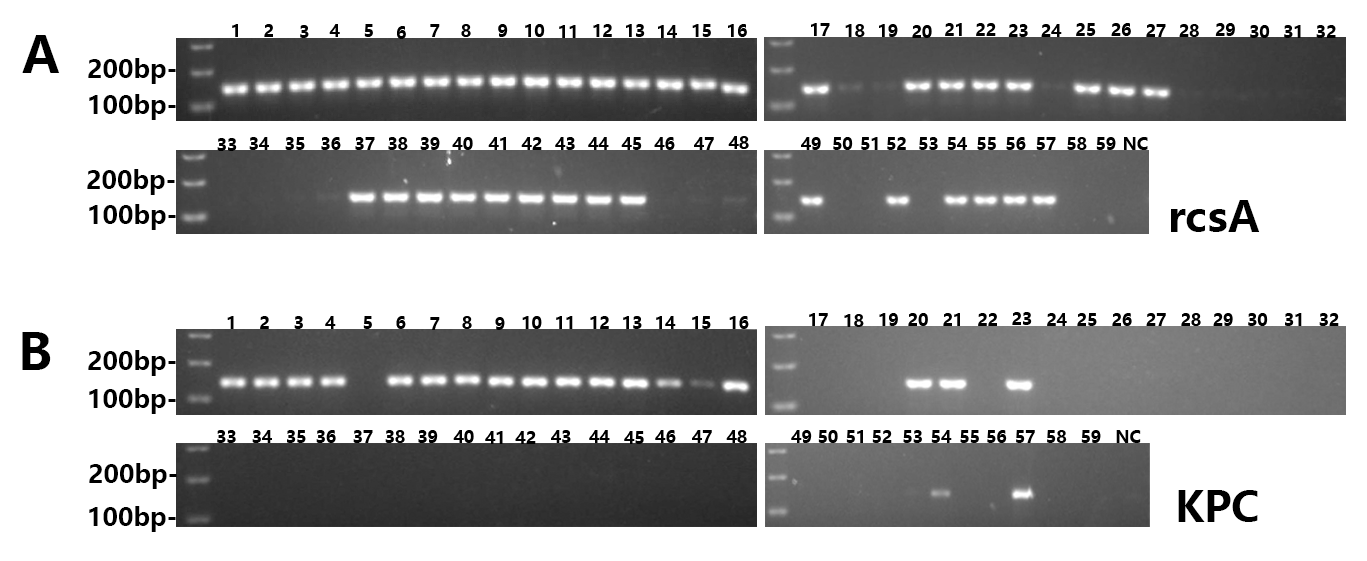
 S2** Results of PCR identification of clinical strains. (A) Results of PCR detection of the rcsA gene. (B) Results of PCR detection of the KPC gene.
